# Usefulness of a One‐step Semi‐deployment Flushing and Stenting Technique in Endoscopic Transpapillary Gallbladder Drainage for Acute Cholecystitis (With Video)

**DOI:** 10.1002/deo2.70163

**Published:** 2025-06-08

**Authors:** Yuki Kawasaki, Jun Ushio, Hisaki Kato, Kazuya Sumi, Yuki Shibata, Norihiro Nomura, Junichi Eguchi, Takayoshi Ito, Haruhiro Inoue

**Affiliations:** ^1^ Department of Digestive Diseases Center Showa University Koto Toyosu Hospital Tokyo Japan

**Keywords:** acute cholecystitis, endoscopic gallbladder stenting, endoscopic retrograde cholangiopancreatography, endoscopic transpapillary gallbladder drainage, one‐step semi‐deployment flushing and stenting technique

## Abstract

**Objectives:**

Endoscopic transpapillary gallbladder stenting (EGBS) has demonstrated high technical and clinical success rates in endoscopic transpapillary gallbladder drainage (ETGBD) for acute cholecystitis. The effectiveness of a 5‐Fr endoscopic naso‐gallbladder drainage (ENGBD) tube for flushing and the internal fistula technique after tube cutting has also been reported. We developed an alternative one‐step semi‐deployed flushing and stenting technique for EGBS using a 7‐Fr pigtail stent that avoids tube resection and evaluated its effectiveness and safety compared with that of endoscopic nasogastric gallbladder drainage.

**Methods:**

We retrospectively evaluated 30 patients who underwent ETGBD for acute cholecystitis between April 2023 and November 2024.

**Results:**

The technical and clinical success rates of the one‐step semi‐deployment flushing and stenting techniques were 95.2% (20/21) and 100% (20/20), respectively. No adverse events were reported. The procedure time did not differ significantly from that of ENGBD.

**Conclusions:**

EGBS using one‐step semi‐deployment flushing and stenting is a simple and effective treatment for acute cholecystitis in patients with ETGBD.

## 1 Introduction

**FIGURE 1:**
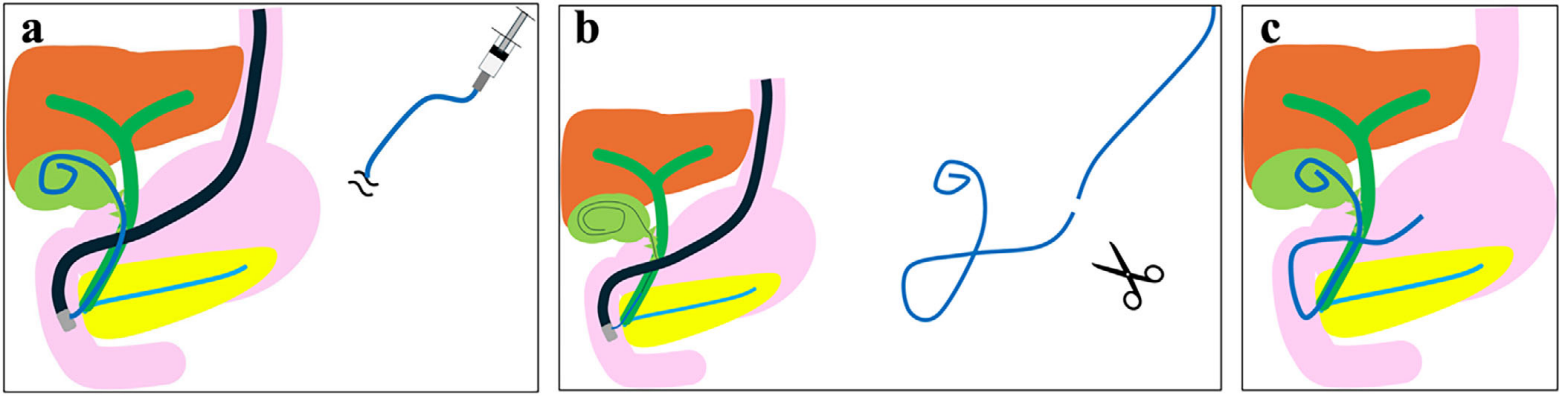
Schema for the flushing and stenting technique using an ENGBD tube: (a) Flushing after ENGBD tube placement. (b) Guide wire replacement and cutting of the ENGBD tubing. (c) ENGBD tube placement as an internal fistula. ENGBD, endoscopic nasogastric bladder drainage.

**FIGURE 2:**
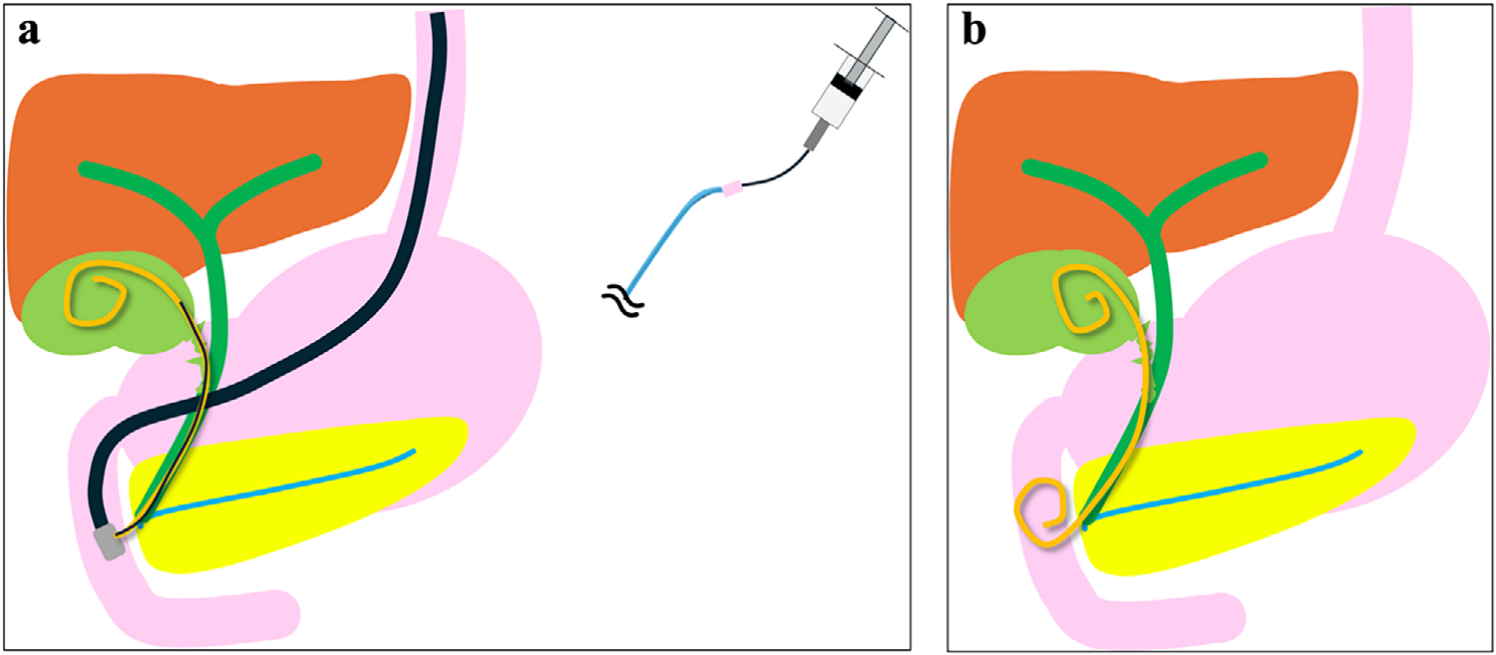
Schema for EGBS using the one‐step semi‐deployment flushing and stenting technique with a 7‐Fr pigtail stent: (a) Flushing after 7‐Fr pigtail stent semi‐deployment. (b) EGBS placement. EGBS, endoscopic transpapillary gallbladder stenting.

Although surgical cholecystectomy is the standard treatment for acute cholecystitis [1, 2, 3], some cases cannot undergo emergency surgery due to patient or facility factors. In such situations, percutaneous transhepatic gallbladder drainage (PTGBD) and endoscopic transpapillary gallbladder drainage (ETGBD) are often performed [4, 5, 6]. PTGBD is the standard drainage method to bridge surgery or palliation [7]; however, patients with ascites and high bleeding risk for cholestatic peritonitis and hemorrhage. Therefore, ETGBD is selected in such patients or those at risk for decreased activities of daily living due to external fistula management [8]. ETGBD includes endoscopic naso‐gallbladder drainage (ENGBD) of an external fistula and endoscopic transpapillary gallbladder stenting (EGBS) of an internal fistula. Randomized controlled trials have demonstrated no significant differences in the technical or clinical success rates between these methods [9, 10]. In a meta‐analysis by Kahan et al., the technical success rates of ENGBD and EGBS were 81% and 85%, while the clinical success rates were 93% and 95%, respectively, in a per‐protocol analysis [11]. Doi et al. developed a new technique involving gallbladder flushing after placement of a 5‐Fr drainage tube and subsequent exchange with EGBS using the same drainage tube to make drainage more secure and eliminate the risk of self‐removal due to an external fistula by ENGBD [12]. A multicenter prospective study in Japan reported technical and clinical success rates of 100% and 96.6%, respectively, for this technique when a guidewire was placed in the gallbladder [13]. Although the technique has shown excellent results, it requires three steps: after flushing with ENGBD drainage tubing (Step 1, Figure 1a), the tube is replaced with a guidewire and cut outside the body for the internal fistula (Step 2, Figure 1b), and the cutting tube is placed as an internal fistula stent (Step 3, Figure 1c). To improve this procedure steps, we developed a one‐step semi‐deployment flushing and stenting technique using an internal fistula stent, flushing (Figure 2a), and placement (Figure 2b) without requiring Step 2.

In this study, we evaluated the usefulness of this one‐step semi‐deployment flushing and stenting technique for ETGBD compared with ENGBD.

## 2 Methods

### 2.1 Patients

**FIGURE 3:**
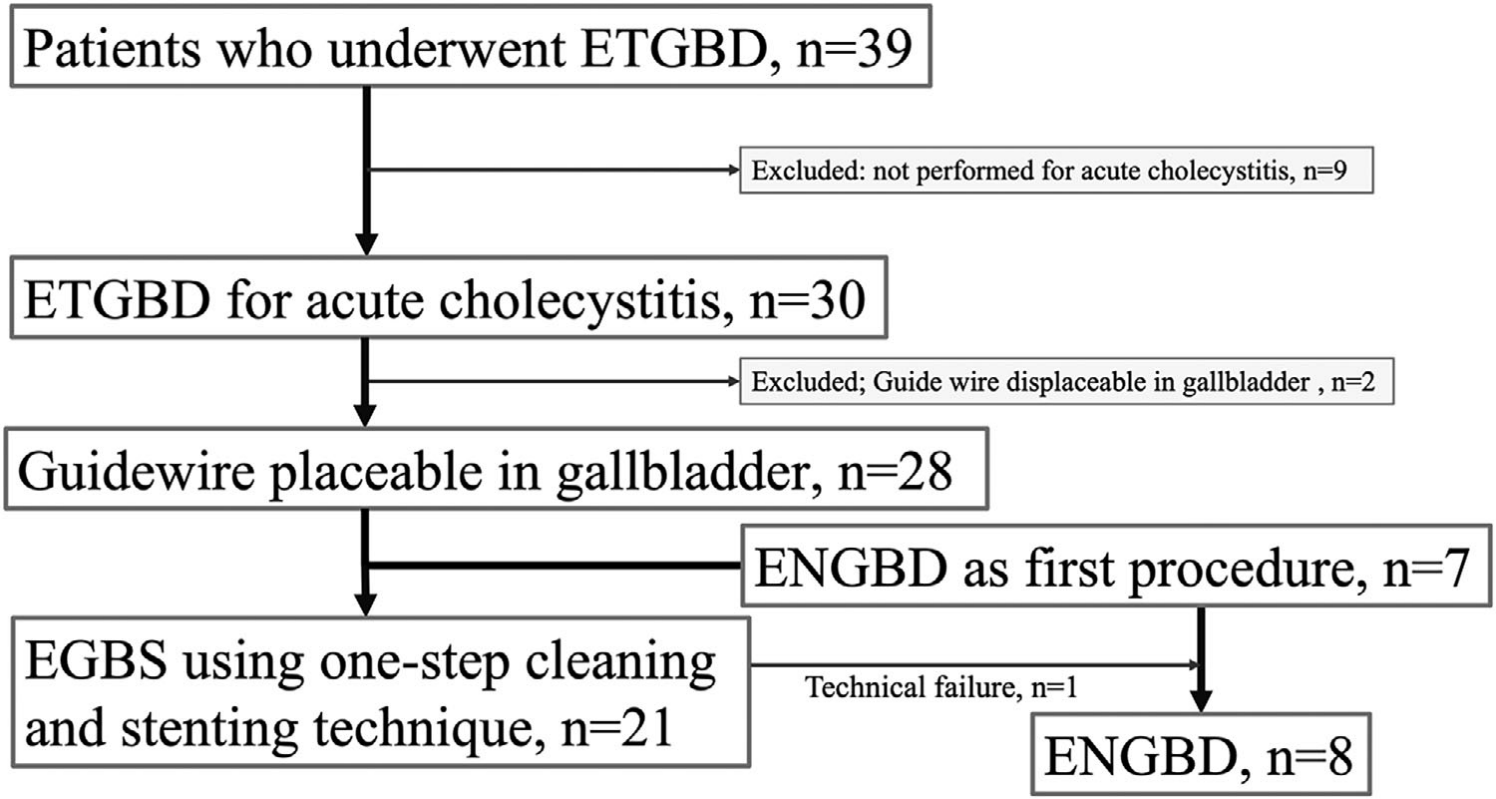
Selection of patients who underwent endoscopic transpapillary gallbladder drainage.

This retrospective analysis included patients who underwent ETGBD for acute cholecystitis between April 2023 and November 2024. The selection process is illustrated in Figure 3. During the study period, 39 patients underwent ETGBD. Of these, nine patients (23%) were excluded because they did not undergo the procedure for acute cholecystitis. Therefore, 30 patients (76.9%) were retrospectively examined. ENGBD was the first choice of transpapillary procedure until June 2023, after which the newly developed one‐step semi‐deployment flushing and stenting technique became the first choice.

### 2.2 One‐step Semi‐deployment Flushing and Stenting Technique

**FIGURE 4:**
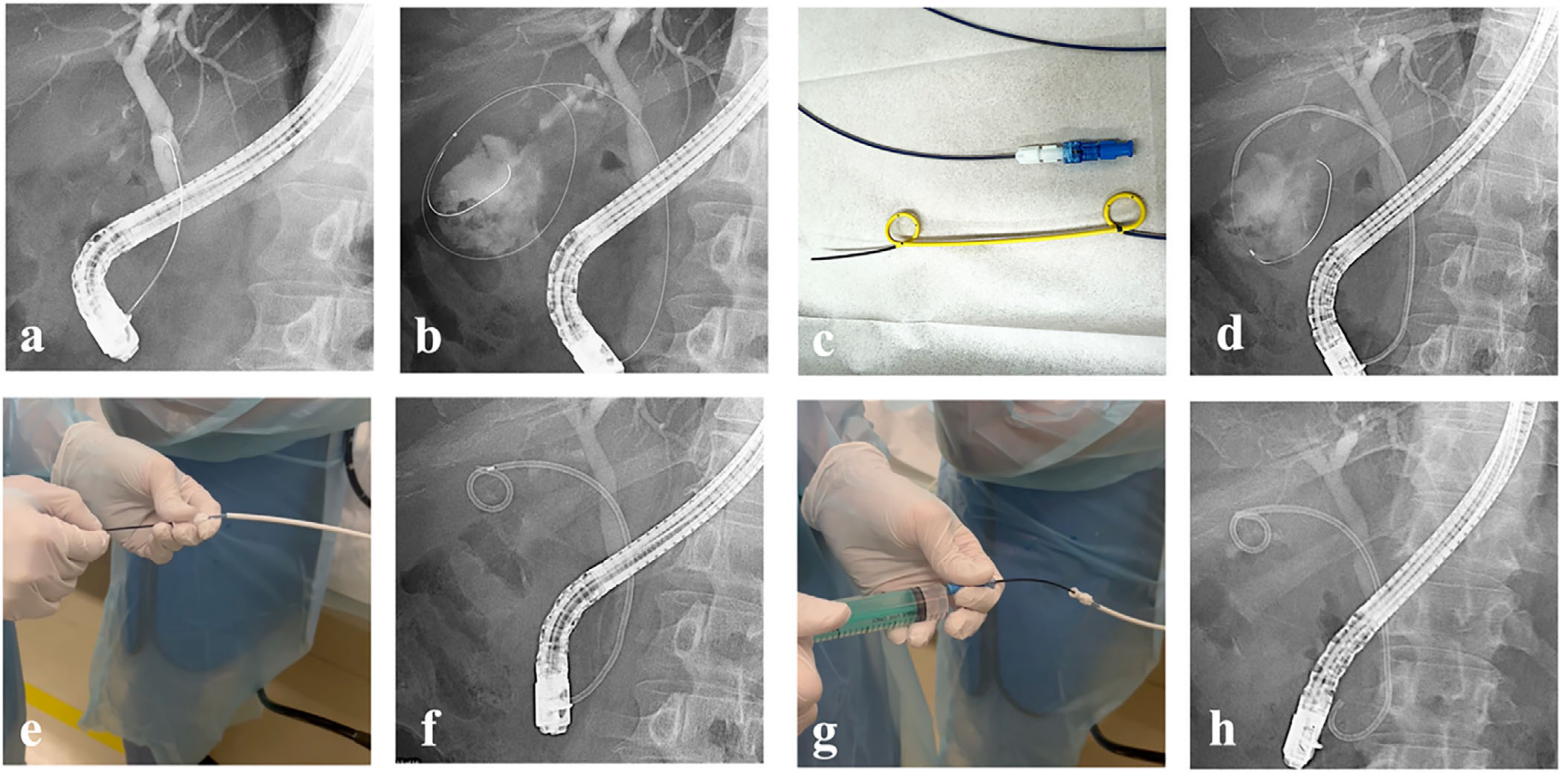
One‐step semi‐deployment flushing and stenting technique: (a) Fluoroscopic image during cystic duct selection. (b) Fluoroscopic image showing guidewire placement in the gallbladder and insertion of the contrast catheter. (c) A 7‐Fr double‐pigtail drainage stent (Through & Pass; Gadelius Medical Co., Ltd., Tokyo, Japan). (d) Fluoroscopic image showing insertion of the 7‐Fr double‐pigtail drainage stent. (e) Half‐deployment of the stent by slightly pulling the inner sheath. (f) Fluoroscopic image showing stent semi‐deployment. (g) Flushing with saline water. (h) Fluoroscopic image showing the fully deployed stent.

ETGBD was performed using a duodenoscope (TJF‐Q290V; Olympus Medical Systems, Tokyo, Japan). After cannulation of the bile duct, the location of the cystic duct was confirmed using cholangiography, and a guidewire was inserted through the cystic duct into the gallbladder (Figure 4a). If the cystic duct could not be confirmed on cholangiography, it was directly sought using a 0.025‐inch, angle‐tip guidewire (VisiGlide; Olympus, Tokyo, Japan). A more flexible hydrophilic guidewire (Radifocus; Terumo, Tokyo, Japan) was used when it was difficult to pass through the cystic duct. After placing a guidewire in the gallbladder, a thin catheter (Shoren; Kaneka Corporation, Osaka, Japan) was inserted into the gallbladder (Figure 4b), and the gallbladder location was confirmed using contrast enhancement. Subsequently, a 7‐Fr double‐pigtail drainage stent (Through & Pass; Gadelius Medical Co., Ltd., Tokyo, Japan) (Figure 4c) was inserted (Figure 4d) and the tip of the pigtail was semi‐developed in the gallbladder (Figure 4e,f). The guidewire was then removed and repeated flushing with saline solution was performed from the distal end of the inner sheath until the bile became sufficiently clear and pneumobilia was confirmed (Figure 4g). The inner sheath was then removed and the stent was completely deployed (Figure 4h). If the 7‐Fr pigtail stent could not be inserted with a 0.025‐inch guidewire, the guidewire was changed to a 0.035‐inch hard‐type (Revowave Seekmaster, Piolax Medical device, Yokohama, Japan). Japan) and the stent was reinserted. When EGBS could not be inserted, the procedure was converted to ENGBD. If ENGBD failed, PTGBD was performed (Video S1).

### 2.3 Outcome Measures

The primary endpoint of the study was the efficacy of the one‐step cleaning and stenting technique. These included technical and clinical success rates and adverse events. The secondary endpoint was the comparison of the technical and clinical success rates and procedure time with those for ENGBD.

### 2.4 Definitions

Technical success of the EGBS one‐step semi‐deployment flushing and stenting technique was defined as the ability to completely insert, flush, and place a stent with a 7‐Fr EGBS after placement of a guidewire in the gallbladder. Clinical success was defined as improvement (or tendency for improvement) in clinical symptoms and laboratory data within three days of the procedure. Adverse events were defined based on the lexicon of endoscopic adverse events outlined by the American Society for Gastrointestinal Endoscopy [14].

### 2.5 Statistical Analysis

Continuous variables, including patient age and procedure duration, are presented as medians and ranges, while categorical variables are presented as proportions. Univariate analyses were performed using χ^2^ or Fisher's exact tests for categorical variables and the Mann–Whitney *U*‐test for continuous variables. Statistical significance was set at p <0.05. All data analyses were performed using JMP Statistics for Windows (version 17.0; SAS Institute Japan Corp., Minato‐ku, Tokyo, Japan).

### 2.6 Ethics Approval

This study was approved by the Showa University Koto Toyosu Hospital Review Board (approval no. 2024‐290‐A) and conformed with the provisions of the Declaration of Helsinki (revised October 2013 in Fortaleza, Brazil).

## 3 Results

### 3.1 Patient Background

The clinical characteristics of the 30 patients (17 men [56.7%] and 13 women [43.3%]) are presented in Table 1. The median patient age was 81 (range: 28–98) years. The cholecystitis severity was mild in eight cases (26.6%) and moderate in 22 cases (73.3%). All patients with severe cholecystitis underwent PTGBD. In 28 patients (93.3%), guidewires were placeable in their gallbladders. Two patients (6.7%) in whom the guidewires could not be placed underwent PTGBD instead.

**TABLE 1:** Clinical characteristics of the study population.

Variables	*n* = 30
Median age, years (range)^a^	81 (28–98)
Male sex, *n* (%)	17 (56.7)
Cholecystitis severity, *n* (%)	
Mild	8 (26.6)
Moderate	22 (73.3)
Severe	0
Guidewire placeable in gallbladder, *n* (%)	28 (93.3)

^a^ Age at endoscopic transpapillary gallbladder drainage placement.

### 3.2 ETGBD Results

The ETGBD results in the study population are presented in Table 2. The EGBS one‐step semi‐deployment flushing and stenting technique was attempted in 21 patients (75%); in one of these patients (4.8%), the 7‐Fr double pigtail stent failed to pass through the cystic duct, and the procedure was converted to ENGBD. Therefore, 29 procedures were performed on 28 patients. ENGBD was performed in eight patients (28.6%), with six and two receiving 6‐Fr and 5‐Fr tube diameters, respectively. The technical success rates were 95.2% (20/21) for the EGBS one‐step semi‐deployment flushing and stenting technique and 100% (8/8) for ENGBD. The clinical success rate was 100% for both techniques. No procedure‐related adverse events, including post‐endoscopic retrograde cholangiopancreatography (post‐ERCP) pancreatitis or stent migration, were observed. The median procedure time was 33 min (range 16–117 min; EGBS one‐step semi‐deployment flushing and stenting technique: 31 min [range 16–117 min]; ENGBD: 35 min [range 23–68 min]). The median flushing time using the one‐step semi‐deployment flushing and stenting technique was 7 min (range 2–9 min). This technique enabled adequate flushing in all cases. The ENGBD was not flushed as it was an external fistula.

**TABLE 2:** Endoscopic transpapillary gallbladder drainage (ETGBD) results for the study population.

Variables	28 Patients, 29 procedures
Drainage method, *n* (%)	
EGBS	21 (75)
ENGBD	8 (28.6)
6‐Fr	6
5‐Fr	2
Technical success rate, % (*n*)	
EGBS	95.2 (20/21)
ENGBD	100 (8/8)
Clinical success rate, % (*n*)	
EGBS	100 (20/20)
ENGBD	100 (8/8)
Adverse events, *n* (%)	
Pancreatitis	0
Migration	0
Median procedure time, min (range)	33 (16–117)
EGBS	31 (16–117)
ENGBD	35 (23–68)
Median EGBS cleaning time, min (range)	7 (2–9)

Abbreviations: EGBS, endoscopic transpapillary gallbladder stenting; ENGBD, endoscopic naso‐gallbladder drainage; ETGBD, endoscopic transpapillary gallbladder drainage.

In the EGBS group, 12 patients were followed after stent placement. During a median follow‐up period of 112 days (range, 24–566 days), no stent migration or recurrence of cholecystitis was observed. Eight patients underwent laparoscopic cholecystectomy, with a median interval from stenting to surgery of 59 days (range, 35–109 days); no adverse effects on the surgical procedure were noted.

In the ENGBD group, internal fistulization with a 7‐Fr pigtail stent was achieved in five patients, with a median time to fistulization of 5 days (range, 4–11 days). In three cases, the external drainage tube was accidentally self‐removed (median time to removal: 3 days [range, 3–7 days]); however, since cholecystitis symptoms had resolved, re‐insertion was not performed. Six patients underwent laparoscopic cholecystectomy, with a median interval to surgery of 58 days (range, 33–99 days). Two elderly patients with self‐removal were managed conservatively and showed no recurrence of cholecystitis during follow‐up periods of 105 and 53 days, respectively.

### 3.3 Comparison of EGBS Using One‐step Flushing and Stenting Techniques With ENGBD

The EGBS one‐step semi‐deployment flushing and stenting technique and ENGBD groups (*p* = 0.01) differed significantly in terms of sex, but were comparable in terms of patient age (*p* = 0.34), cholecystitis severity (*p* = 0.5), technical success (*p* = 0.16), clinical success, median procedure time (*p* = 0.45), and adverse events (Table 3).

**TABLE 3:** Comparison of endoscopic transpapillary gallbladder stenting (EGBS) with endoscopic naso‐gallbladder drainage (ENGBD) using one‐step cleaning and stenting techniques.

Variables	Total *n* = 28	EGBS *n* = 21	ENGBD *n* = 8	*p*‐Value
Median age, years (range)	80 (28–92)	82 (28–92)	74 (46–88)	0.34
Male sex, *n* (%)	17 (56.7)	10 (47.6)	7 (87.5)	0.01
Cholecystitis severity, *n* (%)				0.5
Mild	7 (25)	5	2	
Moderate	21 (75)	16	6	
Technical success, *n* (%)		20 (95.2)	8 (100)	0.16
Clinical success, *n* (%)		21 (100)	8 (100)	–
Median procedure time, min (range)	33 (16–117)	31 (16–117)	35 (23–68)	0.45
From guidewire insertion into the gallbladder to the completion, min (range)		15 (8‐21)	16 (6‐21)	0.29
Adverse events, *n* (%)	0	0	0	–

Furthermore, we compared the time from guidewire insertion into the gallbladder to the completion of the procedure. The median duration was 15 min (range, 8–21 min) in the EGBS group and 16 min (range, 6–21 min) in the ENGBD group, with no statistically significant difference (*p* = 0.29).

## 4 Discussion

The results of this study demonstrated that EGBS using a one‐step cleaning and stenting technique showed sufficient technical and clinical success rates with no increase in procedure time compared with ENGBD.

ENGBD carries the risks of self‐removal, discomfort with the tube remaining in the nose, and the need for multiple procedures to exchange the tube with an internal fistula [15]. The flushing and one‐phase placement technique described by Doi et al. using a 5‐Fr ENGBD tube avoided this risk while enhancing its clinical success as an EGBS [12, 13]. However, 5‐Fr narrow tube stents risk early occlusion. In their meta‐analysis of 7‐Fr stent insertion, Khan et al. reported an EGBS technical success rate of 85% [11], while recent catheters have improved the taper with a guidewire and insertability. However, their definition of technical success included all attempted cases, including those in which guidewire placement into the gallbladder was not achieved. When applying the same definition to our study, the technical success rate was 86.9% (20/23), which is comparable.

In contrast, if only cases with successful guidewire placement into the gallbladder are considered, stent placement failed in just one patient in our cohort, resulting in a high technical success rate of 95.2% (20/21). Thus, our definition focuses on the final step of stent deployment rather than guidewire navigation alone.

Nakahara et al. reported a technique using a 7‐Fr catheter with a side hole to flush the gallbladder, followed by placement of a biliary stent designed for the gallbladder, with a high 7‐Fr catheter insertion rate of 98.9% (175/177) [16]. Doi et al. and Nakahara et al. described a procedure in which only the guidewire is used again after flushing and stenting; however, the present method does not require this replacement procedure, nor does it require a special stent. In addition, we have frequently experienced that flushing using not only the SHOREN catheter employed in this study but also standard catheters within the biliary tract allows for infusion but fails to achieve effective aspiration. In contrast, with this method—possibly due to the presence of side holes—both infusion and aspiration can be performed easily, and adequate flushing was achieved in all cases during this study.

In addition, the procedure times did not differ significantly between EGBS using the one‐step semi‐deployment flushing and stenting technique and ENGBD in this study, although the ENGBD group included few patients. This might be because the median time required for flushing with the one‐step cleaning and stenting technique was 7 min (range, 2–9 min), and the ENGBD group took the same amount of time to place the ENGBD tubes out of the nasal passages. Another factor may be the ease of flushing due to the 7‐Fr stent.

The clinical success rate in this study was 100% in cases in which a stent was placed. Doi et al. and Nakahara et al. also reported 100% clinical success rates when stents were placed after flushing [13, 16], suggesting the effectiveness of flushing the gallbladder for acute cholecystitis. Since both 5‐ and 7‐Fr stents are highly effective, 5‐ or 6‐Fr all‐in‐one narrow pig tail‐type stents may provide higher technical success rates; however, these stents do not exist at present and are awaiting further development.

Distal stent migration may lead to not only cholecystitis progression and recurrence but also pancreatitis and intestinal perforation [17, 18, 19]. In the present study, no stent migration was observed because only short‐term observations were performed (mean follow‐up in non‐surgical cases: 112 days [range, 24–566 days]). However, Nakahara et al. observed stent migration in 25.7% (9/35) of patients with long‐term pigtail stent placement (mean follow‐up period, 180–270 days). Therefore, the risk of migration should be considered in cases involving long‐term pigtail stent placement.

The greatest challenge for ETGBD is the inability to place a guidewire in the gallbladder. Although success rates may increase with surgeon experience [20] and the use of combined intraductal ultrasonography [21] or direct cholangioscopes [22, 23, 24], the results have not been perfect. In recent years, the efficacy of endoscopic ultrasound‐gallbladder drainage (EUS‐GBD) with various stents for acute cholecystitis has also been reported [25, 26]. Lumen‐apposing metal stents (LAMS) have been used in many of these procedures; however, the technique described in the present study may also be useful in EUS‐GBD and other interventional EUS procedures.

The study limitations include the single‐center, retrospective design with few cases. Furthermore, no comparison was made with flushing using the SHOREN catheter. Therefore, a multicenter prospective comparative study using conventional methods is recommended, as well as a comparative study with the flushing group using the SHOREN caterer.

In conclusion, EGBS with a one‐step semi‐deployed flushing and stenting technique is a simple and effective treatment for ETGBD in patients with acute cholecystitis.

## Conflicts of Interest

Haruhiro Inoue was supported by grants from Olympus Corporation and Takeda Pharmaceutical Company and is an advisor for Olympus Corporation and Top Corporation. The other authors declare no conflicts of interest.

## Ethics Statement

This study was approved by the Showa Medical University Koto Toyosu Hospital Review Board (approval no. 2024‐290‐A) and conformed with the provisions of the Declaration of Helsinki (revised October 2013 in Fortaleza, Brazil).

## Consent

N/A.

## Clinical Trial Registration

N/A.

## Supporting information




**VIDEO S1** One‐step semi‐deployment flushing and stenting technique in endoscopic transpapillary gallbladder drainage.
